# Decubitus ulcers are associated with prolonged length of stay in critically ill patients

**DOI:** 10.1186/cc12403

**Published:** 2013-03-19

**Authors:** WT McGee, BH Nathanson, E Lederman, TL Higgins

**Affiliations:** 1 Baystate Medical Center, Springfield, MA, USA; 2 OptiStatim LLC, Longmeadow, MA, USA

## Introduction

How decubitus ulcers (DU) diagnosed on admission to the ICU affect resource use is unknown. We hypothesize that this easily identifiable clinical finding would be associated with longer hospitalizations.

## Methods

Clinical assessment was used to identify patients with DU on admission to the ICU. The association of DU on hospital and ICU length of stay (LOS) was assessed with generalized linear models that adjusted for ICU admission source, age, APACHE IV score, diabetes, sepsis, hepatic failure, dialysis and mechanical ventilation.

## Results

DU was present on admission in 180 of 2,723 (6.6%) during a 19-month period in 2011 to 2012 at a single teaching hospital in New England. Patients with decubiti were sicker on admission (mean (SD) APACHE IV 80.2 (28.2) vs. 58.7 (30.7), *P *0.001), more likely to be age 65 or older (58.9% vs. 41.8%, *P *0.001) and require more organ supportive therapies (Table 1). DU patients had a higher in-hospital mortality rate (32.2% vs. 18.3%, *P *0.001) and longer mean ICU and hospital LOS (4.9 vs. 3.3 days and 15.6 vs. 10.5 days, respectively (*P *0.001)). After multivariate adjustment, DU patients stayed 3.2 days, 95% CI (1.6, 4.8) longer in the hospital than non-DU patients and stayed 2.5 days longer, 95% CI (0.7, 0.43) among survivors. DU patients had longer ICU stays (1.3 days, 95% CI (0.3, 1.3)) in general but were statistically similar among survivors.

**Table 1:** 

	Admitted	Admitted	
	with	without	
	decubitus	decubitus	*P*
Variable	(*n *= 180)	(*n *= 2,543)	value
Age 65 or older	106 (58.9%)	1,062 (41.8%)	<0.001
APACHE IV score on admission	80.2 (28.2%)	58.7 (30.7)	<0.001
Diabetes	53 (29.4%)	420 (16.5%)	<0.001
Dialysis	16 (8.9%)	58 (2.3%)	<0.001
Hepatic failure	3 (1.7%)	56 (2.2%)	1.000
Mechanically ventilated during	123 (68.3%)	1,457 (57.3%)	
hospitalization			
Admission source to the ICU			
Direct admit	10 (5.6%)	154 (6.1%)	
ER	84 (46.7%)	1,206 (47.4%)	
Floor	25 (13.9%)	214 (8.4%)	0.017
OR	19 (10.6%)	459 (18.1%)	
Other location	42 (23.3%)	510 (20.1%)	
Outcomes			
ICU LOS (days)	4.9 (4.9)	3.3 (4.7)	<0.001
Hospital LOS (days)	15.6 (20.7)	10.5 (11.1)	<0.001
ICU mortality	30 (16.7%)	308 (12.1%)	0.073
Hospital mortality	58 (32.2%)	464 (18.3%)	<0.001
Readmission rate for	20 out of 112	173 out of 2,079	0.002
survivors	(16.4%)	(8.3%)	

## Conclusion

The presence of decubiti on admission to the ICU is associated with longer hospitalizations even after adjusting for age, acuity, and organ supportive therapies. DU on admission to ICU provide a unique, unambiguous marker of increased resource utilization.

